# Zhengtian Capsule versus flunarizine in patients with migraine: a multi-center, double-blind, double-dummy, randomized controlled, non-inferior clinical trial

**DOI:** 10.1186/s12906-016-1321-8

**Published:** 2016-09-13

**Authors:** Kegang Cao, Fang Han, Anji Lin, Wenming Yang, Jianjun Zhao, Hui Zhang, Yanbing Ding, Wei Xie, Yinping Xu, Tingmin Yu, Xinzhi Wang, Xiaosu Yang, Jiying Zhou, Qun Hou, Lihua Yu, Ying Gao

**Affiliations:** 1 Department of Neurology, Dongzhimen Hospital, Beijing University of Chinese Medicine, No.5 Haiyun Cang, Dong Cheng District, Beijing, China; 2 Department of Neurology, Xiamen Hospital of Traditional Chinese Medicine, No.1739 Xianyue Road, Xiamen, China; 3 Department of Neurology, The First Subsidiary Hospital of Anhui College of Traditional Chinese Medicine, No.117 Meishan Road, Hefei, China; 4 Department of Neurology, The Affiliated Hospital to Changchun University of Chinese Medicine, No.1478 Gongnong Avenue, Chaoyang District, Changchun, China; 5 Department of Traditional Chinese Medicine, the Central Hospital of Xuhui District, No.966 Huaihai Road, Shanghai, China; 6 Department of Neurology, Hubei Hospital of Traditional Chinese Medicine, No.4 Huayuan Shan, Wu Chang District, Wuhan, China; 7 Department of Traditional Chinese Medicine, Nanfang Hospital of Southern Medical University, No.1838 Guangzhou Road north, Guangzhou, China; 8 Department of Acupuncture, Beijing Pinggu District Hospital of Traditional Chinese Medicine, No.6 Pingxiang Road, Pinggu district, Beijing, China; 9 Department of Neurology, the Second Hospital of Jilin University, No.218 Ziqiang Street, Nanguan District, Changchun, China; 10 Department of Neurology, the First Affiliated Hospital of Henan University of Traditional Chinese Medicine, No.19 Renmin Road, Zhengzhou, China; 11 Department of Neurology, the Xiangya Hospital Central South University, No.87 Xiangya Road, Changsha, Hunan Province China; 12 Department of Neurology, the First Affiliated Hospital of Chongqing University of Traditional Chinese Medicine, No.1 Youyi Road, Yuanjia Gang, Yuzhong District, Chongqing, China; 13 Department of Neurology, Zhejiang Hospital of Traditional Chinese Medicine, No.54 Youdian Road, Hangzhou, China; 14 Department of Emergency Medicine, the Third Hospital affiliated with Beijing University of Traditional Chinese Medicine, Beijing, China

**Keywords:** Zhengtian Capsule, Flunarizine, Migraine, Randomized controlled trial, Non-inferior

## Abstract

**Background:**

The primary objective of this study was to assess whether Zhengtian Capsule was non-inferior to flunarizine in efficacy and safety profile for prevention of migraine in adults.

**Methods:**

This was a double-dummy, double-blind, multicenter, positive drug (flunarizine), parallel randomized controlled, non-inferior clinical trial. Patients (*n* = 360) were randomized in a 1:1 to receive either Zhengtian Capsule or flunarizine, including 12 weeks’ intervention and 4 weeks’ follow-up. The primary outcome measure was responder rate (defined as the percentage of subjects in a treatment group with 50 % or greater reduction in attack frequency during treatment compared with the baseline period). The secondary outcome measures included migraine attack frequency, the number of migraine days, pain evaluated by visual analogue scale (VAS) score, duration of migraine attacks, the times of using analgesics, patient-reported outcome (PRO) measure of migraine and the scores of short-form 36 Health Survey Scale (SF-36). Weight variation in both groups was also evaluated. Adverse events were monitored throughout the trial.

**Results:**

Zhengtian Capsule was non-inferior to flunarizine in responder rate at week 12 and follow-up period (*P* = 0.002, *P* < 0.001). There was fewer migraine days in Zhengtian Capsule group at follow-up period compared with flunarizine (*P* = 0.001). For the total duration of migraine attacks, there was significant group difference at week 4 which favored the control group (*P* = 0.009). For the total score of PRO scale, there was statistical difference between the two groups at follow-up period (*P* = 0.021). There were also group differences between the two groups in the dimensions of somatization symptoms at week 4 (*P* = 0.022) and functional status at week 12 and follow-up period (*P* < 0.001, *P* < 0.001). However, there were no significant differences between the two groups in migraine attack frequency, VAS scores reduction, consumption of acute pain drugs and the dimension scores of SF-36 at any time interval of the treatment period (*P* > 0.05). No severe adverse events occurred in the trial. Flunarizine was found associated with a weight gain.

**Conclusion:**

Zhengtian Capsule was non-inferior to flunarizine with regard to the primary endpoint. In addition, it could reduce migraine days and improve the functional status and somatization symptoms of migraine patients with good safety profile.

**Trial registration:**

This trial was registered at Chinese Clinical Trial Register (ChiCTR), ChiCTR-TRC-13004412.

## Background

Migraine is a common disabling primary headache disorder characterized by recurrent unilateral location, throbbing quality, moderate or severe intensity, associated with symptoms such as nausea, vomiting, photophobia and phonophobia, usually aggravated by routine physical activities [1]. According to the Global Burden of Disease Survey 2010 (GBD2010), migraine was estimated a global prevalence of 14.7 %, ranked as third most common diseases in the world in both males and females [2]. Generally, it has been reported that female migraine sufferers tend to outnumber male sufferers by nearly 3 to 1 [3–5]. Migraine is burdensome and costly. On account of substantial impairment on work or school productivity, it negatively impacts human capital accumulation and brings about a heavy socioeconomic burden [6–8]. Migraine is also a disease that contributes to major disability thus leading to low life quality. It was ranked as the seventh-highest specific cause of disability worldwide [2, 9]. In addition, it is associated with a number of comorbidities, including psychiatric disease, sleep disturbances, stroke and other chronic pain disorders [10–12]. The status quo in China is similar to the world average [13, 14]. Due to its high prevalence and disabling feature, migraine has become an important target issue for public health interventions.

Migraine is divided into acute episode and chronic remission period. Accordingly, the treatment is divided into abortive treatment and preventive treatment. The former intends to return the patient to full function within 2 h, whereas, the latter with a goal of improving the health-related quality of life in patients with migraine. For acute treatment, there are specific and nonspecific treatment methods. Triptans and dihydroergotamine are effective specific medications, nonsteroidal anti-inflammatory drugs, opioids and barbitals are effective nonspecific medications [15]. For the preventive treatment, there are angiotensin receptor blockers (ARB), angiotensin converting enzyme inhibitors (ACEI), antiepileptic drugs (AEDs), antidepressant drugs, calcium channel blockers (CCB), beta-receptor blocker, etc. However, there exists some side effects which inhibit the widely use of these drugs [16, 17]. In this trial we use flunarizine as the positive comparator drug because it is widely used for migraine with proven efficacy and safety. Zhengtian Capsule is a dosage form modified from Zhengtian Pill, which is a Chinese Patent medicine proved effectively in treating migraine in previous studies [18, 19]. It is composed of 15 herbal compositions: Uncariae Ramulus Cum Uncis(Gou Teng), Ephedrae Herba(Ma Huang), Asari Radix Et Rhizoma(Xi Xin), Aconiti Lateralis Radix Praeparata(Fu Pian), Paeoniae Radix Alba(Bai Shao), Persicae Semen(Tao Ren), Cartham Flos(Hong Hua), Rehmanniae Radix(Di Huang), Angelicae Sinensis Radix(Dang Gui), Chuanxiong Rhizoma(Chuanxiong), Notopterygh Rhizoma et Radix(Qiang Huo), Angelicae Pubescentis Radix(Du Huo), Saposhnikoviae Radix(Fang Feng), Spatholobi Caulus(Ji Xue Teng), Angelicae Dahuricae Radix(Bai Zhi). Studies on Zhengtian Capsule with a multicenter, double blind, randomized controlled trial are rarely known. Hence, we conducted a multi-center, double-blind, double-dummy, randomized controlled, non-inferior clinical trial to determine whether or not Zhengtian Capsule was non-inferior in effectiveness and safety to flunarizine in the prophylaxis of migraine.

## Methods

### Study design

A multicenter, prospective, double-blind, double-dummy, positive drug parallel controlled, non-inferior clinical trial was conducted in our study. The study was carried out on 360 patients with migraine in 13 participating centers simultaneously. Since Zhengtian Capsule was previously reported rarely, the sample calculation of the study was determined in reference of Zhengtian Pill. The previous study showed that the responder rate of migraine attacks was 63.3 % in Zhengtian Pill and 52 % in flunarizine. We designed a non-inferiority testing. The sample size was estimated using standard methods for test of non-inferiority. Non-inferiority margin is - 5 %, alpha = 0.05, beta = 0.10. The computational formula was as follows:

$$ \begin{array}{c}n=\frac{{\left({z}_{1-\alpha }+{z}_{1-\beta}\right)}^2\times \left[{P}_1\left(1-{P}_1\right)+{P}_2\left(1-{P}_2\right)\right]}{{\left(\varepsilon -\delta \right)}^2}\\ {}=\frac{{\left(1.65+1.28\right)}^2\times \left[0.63\left(1-0.63\right)+0.52\left(1-0.52\right)\right]}{{\left(0.113-\left(-0.05\right)\right)}^2}\\ {}\approx 156\end{array} $$

To allow for a maximum drop-out rate of up to 15 %, 360 individuals should be enrolled in this study. We allocated a proportion of the two groups (1:1) and determined 180 patients for each group. There were a 3-month well documented retrospective history and a run-in period of 4 weeks without treatment. Then migraineurs who met the inclusion criteria were assigned to the experimental group and the control group randomly. The experimental group were treated with Zhengtian Capsule (0.9 g, three times a day) and flunarizine simulation (5 mg, quaque nocte). The control group were treated with flunarizine (5 mg, q.n) and Zhengtian Capsule simulation (0.9 g, tid). All the participants then underwent a therapeutic course of 12-week and 4-week of follow-up. There were totally five visits for each eligible patient conducted every 4-week interval: At baseline, week 4, week 8, week 12 and follow-up period respectively. Ibuprofen and other analgesic drugs were permitted during the process if necessary which were recorded in detail. Headache diary was handed out to each included patient, they were instructed to note the characteristics of the migraine attacks such as headache severity, functional disability, associated symptoms such as nausea, vomiting, photophobia, and phonophobia. The protocol and informed consent were reviewed and approved by the Ethics Committee of the Affiliated Dongzhimen Hospital of Beijing University of Chinese Medicine before commencing the trial. This study protocol was in accordance with the Declaration of Helsinki and Good Clinical Practice (GCP) ethical guidelines. Written informed consent was obtained from each subject prior to participation. The randomization sequence was computer generated in a 1:1 ratio by an independent statistician using SAS statistical software. There were accordant drug codes. All of the trial drugs were dispensed sequentially by a specified drug administrator in a separate reception room according to the sequence of inclusion of eligible patients. There were also sealed envelopes which served as emergency envelopes, when there were severe adverse events investigators would know which drug patients had received. All researchers, participants and statisticians were masked to treatment allocation throughout the trial.

### Setting and participants

Patients were recruited through advertising and outpatient departments of the 13 participating hospitals across China and through advertising. Four hundred eighteen patients were screened and 360 patients were randomized to treatment. The assessment was performed by principle investigators of the participating hospitals. The 13 participating hospitals were as follows: Dongzhimen Hospital affiliated to Beijing University of Chinese Medicine, Xiamen Hospital of Traditional Chinese Medicine, the First Subsidiary Hospital of Anhui College of Traditional Chinese Medicine, the Affiliated Hospital to Changchun University of Chinese Medicine, the Central Hospital of Xuhui District, Hubei Hospital of Traditional Chinese Medicine, Nanfang Hospital of Southern Medical University, Beijing Pinggu District Hospital of Traditional Chinese Medicine, the Second Hospital of Jilin University, the First Affiliated Hospital of Henan University of Traditional Chinese Medicine, the Xiangya Hospital Central South University, the First Affiliated Hospital of Chongqing University of Traditional Chinese Medicine, Zhejiang Hospital of Traditional Chinese Medicine. All the patients had blood routine, urine routine, liver and renal function tests, electrocardiogram examinations, myocardial enzyme, myocardial injury markers, CT or MRI to exclude severe accompanying conditions before entrance to the trial.

### Diagnostic criteria

Migraine without aura (MO) or with a typical aura (MA) both fulfilled the second edition of International Classification of Headache Disorders (ICHD-II) diagnostic criteria for migraine strictly [20].

### Inclusion criteria

A subject would be eligible for inclusion in the trial only if all the following criteria were fulfilled at baseline: (1) Diagnosed as MO or MA according to the diagnostic criteria specified by ICHD-II [20]. (2) Age of first onset ≤ 50 years old. (3) The patients had at least a 12-month history of migraine with or without aura as defined by the criteria of the 2004 International Headache Society. (4) Average migraine attacks per month were between 2 and 6 (including two times and six times) at baseline period. (5) Age between 18 and 65 years old.

### Exclusion criteria

A subject would not be eligible for inclusion in the trial if any of the following criteria applied at baseline: (1) Analgesics were used for acute headache >10 times per month. (2) For the previous 3 months before inclusion, migraine prevention drugs was taken, such as beta blockers, calcium channel blockers, anti-epileptic drugs, antidepressants and 5-HT receptor blockers, etc. (3) Alcohol or other drug abusers. (4) Combined with severe primary diseases such as cardiovascular, cerebrovascular, liver, kidney, hematopoietic system disease, etc. (5) Psychiatric patients. (6) Allergic to the trial drugs. (7) Pregnancy and lactation.

### Interventions

Participants in the experimental group were given Zhengtian Capsule and flunarizine simulation, while the control group were given flunarizine and Zhengtian Capsule simulation. The active drugs and simulations had identical appearance and were indistinguishable in taste, color, appearance and smell. Both of them were issued in a sealed box and provided by Hua Run San-Jiu Pharmaceutical Co.LTD. Zhengtian Capsule was taken 0.9 g at a time, three times a day after meal while flunarizine was taken 5 mg, quaque nocte. Ibuprofen and other analgesic drugs were permitted to use when necessary and should be well recorded. With other diseases such as infection, hypertension and diabetes, concomitant therapies that were allowed or on a restricted basis should be specified.

### Outcome measures

The primary outcome measure was responder rate (defined as the percentage of subjects in a treatment group with 50 % or greater reduction in attack frequency during treatment compared with the baseline period) [21]. Migraine attack temporarily relieved due to sleep or treatment relapsed within 48 h was defined as one single attack. Forty-eight hours of freedom between attacks of migraine permits identification of individual attacks.

The secondary outcome measures were migraine attack frequency, the number of migraine days, intensity for pain evaluated by VAS score, duration of migraine attacks, the times of using analgesics, PRO sacle of migraine and the SF-36 questionnaire. Weight changes were also measured. The VAS is a 10 cm continuous horizontal line that measures the severity of headache, it is a quantitative index for pain. Make the patients point out the number that can mostly represent the pain of his headache. Zero stands for no headache, ten stands for extreme headache. Score range 0 ∼ 4 stands for mild headache, score range 4 ∼ 7 stands for moderate headache, score range 7 ∼ 10 stands for severe headache [22]. The duration of each attack refers to the time from migraine onset to vanish, if the patient goes to sleep in pain, the time of vanishing is when he wakes up. Patient reported outcome (PRO) is a modern scientific approach widely used in evaluating outcomes of clinical trials based on patient centred evidence [23–25]. A PRO is defined as any report of the status of a patient’s health condition that comes directly from the patient, without interpretation of the patient’s response by a clinician or anyone else [26]. It highlights the importance of assessing the effectiveness of health care from the patients’ perspective.

Since there was no PRO sacle validated for migraine patients. Our previous study established a PRO sacle for remission period migraine patients with comprehensive contents and reasonable structure [27]. The four domains are headache, somatization symptoms, psychological state and functional status. It has been proved to imply treatment effect from multiple dimensions and with good reliability, validity and reaction degree [28, 29]. The PRO sacle also has a good connotation of traditional Chinese medicine with the concept of the holism of body and spirit as one of the basic Chinese medical theories. It has become a good complement to migraine clinical efficacy evaluation system and should not be ignored.

The SF-36 questionnaire assesses Health Related Quality Of Life in eight domains: Mental Health (MH), Role Emotional (RE), Social Functioning (SF), Vitality (VT), General Health (GH), Body Pain (BP), Role Physical (RP), Physical Functioning (PF). There are also two scales for mental and physical health, Mental Component Summary (MCS) score and Physical Component Summary (PCS) score.

In addition, the blood routine, urine routine, liver and kidney function tests, electrocardiogram, myocardial enzyme and myocardial injury markers were evaluated at the baseline and the treatment ended as safety evaluation endpoints. During the study, all adverse events were monitored and recorded on the case report form (CRF) including time of onset and resolution, severity and the relationship between adverse events and drugs analyzed by investigators.

### Statistical methods

SAS (Statistical package version 9.2) was used for Statistical analysis, which were prespecified using both per-protocol set (PPS) and full analysis set (FAS). PPS analyses were based on data from patients who had good compliance to complete the trial according to the criteria of protocol and who had taken 80–120 % amount of trial drugs. FAS analyses took the principle of intention-to-treat which were based on data from all the randomized patients who took at least one trial drug and the last observation carried forward (LOCF) approach was used to impute missing data. FAS is the main analysis set in this trial.

In addition, responder rate was compared between the two groups using non-inferior test, with non-inferiority margin - 0.5. For the changes in migraine attack frequency, the number of migraine days, duration of migraine attacks, the times of using analgesics, VAS scores, PRO scores, weight changes and SF-36 dimension scores, analysis of covariance (ANCOVA) with baseline variables as covariates were used to compare the differences between the two groups at different time points. The level of significance was set at 0.05, if *P* < 0.05, there were statistical differences.

## Results

**Fig. 1:**
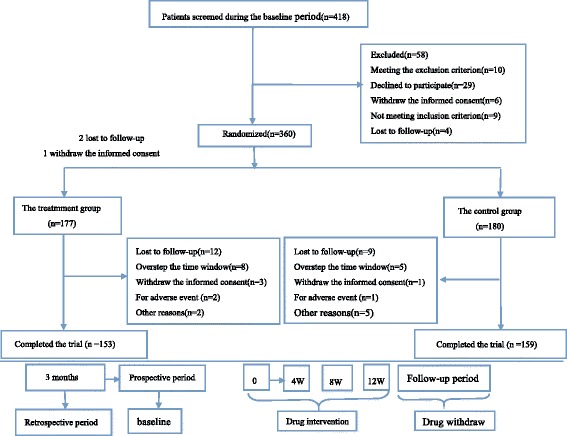
Flow diagram of the study progress about enrollment, randomization, intervention, and completion of the trial

Of all the 360 randomized patients, 357 of their data were included in FAS analyses (intention-to-treat analyses). There were two lost to follow up and one withdrew the informed consent in the treatment group. Therefore, 177 of 180 patients in treatment group and all patients in control group were included into FAS. In comparison, 312 patients were put into PPS, 153 from the treatment group and 159 from the control group. In the process of the entire study, 48 patients dropped out, a rate of 13.33 % (27 from the treatment group 15 %, 21 from the control group 11.67 %). Among these, 21 patients lost to follow-up (12 from the treatment group, nine from the control group), four patients withdrew the informed consent (three from the treatment group, one from the control group), three patients dropped out for adverse events (two from the treatment group, one from the control group), 13 patients dropped out because of overstepping the time window (eight from the treatment group, five from the control group), seven dropped out for other reasons. The reasons for the dropouts are detailed in Fig. 1.

### Characteristics of demography and baseline

The demographic and baseline clinical characteristics of the two groups were comparable at baseline as shown in Table 1. There were no statistical significances in FAS between the two groups (*P* > 0.05).

**Table 1:** Baseline characteristics of migraine patients (FAS)

Parameters	Treatment group (*n* = 177)	Control group (*n* = 180)	*P* value
sex(Male/Female)	54/123	63/117	0.366
Age(year)	39.70 ± 12.81	38.99 ± 11.56	0.654
weight	59.74 ± 9.22	60.94 ± 10.07	0.301
Course (months)	95.76 ± 86.82	91.61 ± 81.87	0.695
Migraine days	4.02 ± 2.16	4.09 ± 1.73	0.181
Migraine attacks	3.37 ± 1.16	3.53 ± 1.11	0.099
Total duration(h)	31.87 ± 36.47	30.26 ± 27.90	0.936
VAS scores	5.63 ± 1.31	5.63 ± 1.27	0.661
Analgesic consumption(times)	1.34 ± 1.66	1.35 ± 1.87	0.726
Total scores of PRO	34.34 ± 5.82	34.94 ± 5.63	0.189
Headache	12.50 ± 1.983	12.71 ± 1.89	0.237
Somatization symptoms	10.53 ± 2.47	10.50 ± 2.51	0.910
Psychological state	4.71 ± 1.46	4.89 ± 1.49	0.195
Functional status	5.77 ± 1.51	5.92 ± 1.49	0.348
PF	92.91 ± 7.07	92.97 ± 8.08	0.527
RP	63.07 ± 37.51	64.56 ± 37.56	0.257
BP	53.71 ± 13.78	52.97 ± 13.98	0.636
GH	60.91 ± 16.93	61.86 ± 17.19	0.531
VT	72.52 ± 13.87	72.31 ± 14.71	0.781
SF	77.07 ± 13.18	75.24 ± 14.56	0.630
RE	70.42 ± 36.22	64.13 ± 39.73	0.371
MH	72.93 ± 14.41	71.39 ± 15.87	0.766
MCS	73.23 ± 14.83	70.77 ± 17.60	0.620
PCS	67.65 ± 14.31	68.09 ± 14.68	0.596

### Responder rate

The responder rates in the treatment group and control group were 21.47 % VS 21.11 % after 4 weeks (*P* = 0.108), 34.46 % VS 38.89 % after 8 weeks (*P* = 0.455), 59.89 % VS 49.44 % after 12 weeks (*P* = 0.002) and 54.80 % VS 38.33 % at follow-up period (*P* < 0.001). After 12 weeks’ treatment and during follow-up period, Zhengtian Capsule was non-inferior to flunarizine (Table 2).

**Table 2:** Comparison of responder rate (FAS)

Responder rate	Treatment group (*n* = 177)	Control group (*n* = 180)	*P* value
4 W	38(21.47 %)	38(21.11 %)	0.108
8 W	61(34.46 %)	70(38.89 %)	0.455
12 W	106(59.89 %)	89(49.44 %)	0.002*
Follow-up period	97(54.80 %)	69(38.33 %)	<0.001*

Note: Non-inferiority margin was −0.5

*P < 0.05 for data comparison between groups after treatment

### Migraine attacks

Migraine attacks have significantly decreased in the treatment group from 3.37 ± 1.16 at baseline to 1.37 ± 1.30 during follow-up period, whereas from 3.53 ± 1.11 to 1.55 ± 1.30 in the control group. However, no group differences were seen at any time points (Table 3).

**Table 3:** Differences from baseline in efficacy variables at different time points (FAS)

Variable	Treatment group (*n* = 177)	Control group (*n* = 180)	*P* value
Migraine attacks			
4 W	2.66 ± 1.40	2.66 ± 1.20	0.864
8 W	2.04 ± 1.30	1.93 ± 1.19	0.153
12 W	1.57 ± 1.46	1.42 ± 1.12	0.460
Follow-up period	1.37 ± 1.30	1.55 ± 1.30	0.398
Migraine days			
4w	2.98 ± 1.74	2.91 ± 1.41	0.994
8w	2.25 ± 1.72	2.13 ± 1.42	0.588
12 w	1.73 ± 1.78	1.58 ± 1.34	0.093
Follow-up period	1.46 ± 1.55	1.68 ± 1.50	0.001*
VAS scores			
4w	4.94 ± 1.26	4.79 ± 1.14	0.990
8w	4.34 ± 1.31	4.22 ± 1.23	0.404
12 w	3.58 ± 1.05	3.26 ± 1.47	0.141
Follow-up period	3.87 ± 1.25	3.85 ± 1.41	0.852
Total Duration(h)			
4w	19.70 ± 19.69	19.18 ± 19.24	0.009*
8w	14.68 ± 21.83	12.41 ± 14.41	0.753
12 w	11.71 ± 24.83	8.69 ± 12.89	0.526
Follow-up period	7.55 ± 13.76	7.31 ± 11.74	0.140
Analgesic consumption (times)			
4w	0.96 ± 1.30	1.01 ± 1.59	0.229
8w	0.86 ± 1.65	0.76 ± 1.42	0.862
12w	0.70 ± 1.52	0.51 ± 1.07	0.360
Follow-up period	0.51 ± 1.37	0.41 ± 1.29	0.652

Note: *P < 0.05 for data comparison between groups after treatment

### Number of migraine days

As compared to baseline, migraine days decreased throughout the study period in both groups. The difference in the number of migraine days between the two groups was significant at follow-up period (*P* = 0.001) (Table 3).

### VAS scores

After treatment, the VAS scores in the two groups declined month by month compared with the baseline. During the treatment course, the VAS scores decreased from 5.63 ± 1.31 to 3.87 ± 1.25 throughout the study period in the treatment group, whereas from 5.63 ± 1.27 to 3.85 ± 1.41 in the control group. However, there were no statistical differences between the two groups at any 4-week of time interval (Table 3).

### Total duration of migraine attacks

There was a significant decrease in total duration of migraine attacks per 4 weeks in both groups, and there was significant group difference at week 4 which favored the control group (*P* = 0.009) (Table 3).

### Analgesic consumption

In both groups, frequency of using acute pain drugs at each time point was markedly reduced compared to baseline. However, there were no significant differences between the two groups at week 4, week 8, week 12 and follow-up period (*P* > 0.05) (Table 3).

### The scores of patient reported outcome (PRO) scale of migraine

During the study period, the total scores of PRO were descending gradually in both groups. At follow-up period, there was statistical difference between the two groups (*P* = 0.021). For the dimension score of functional status, there were group differences between the two groups at week 12 and follow-up period (*P* < 0.001, *P* < 0.001). For the dimension score of somatization symptoms, there was group difference at week 4 after treatment (*P* = 0.022) (Table 4).

**Table 4:** Comparison of the scores of PRO for migraine (FAS)

	Treatment group (*n* = 177)	Control group (*n* = 180)	*P* values
Total score			
4w	29.68 ± 5.87	30.18 ± 5.86	0.767
8w	26.97 ± 6.72	26.79 ± 7.26	0.455
12 w	22.90 ± 6.63	23.80 ± 7.27	0.301
Follow-up period	21.61 ± 6.47	23.41 ± 6.80	0.021*
Headache			
4w	10.88 ± 2.07	11.05 ± 2.24	0.725
8w	9.56 ± 2.62	9.52 ± 2.76	0.643
12 w	8.54 ± 2.78	8.23 ± 2.88	0.246
Follow-up period	7.95 ± 2.73	8.31 ± 2.98	0.283
Somatization symptoms			
4w	9.46 ± 2.66	9.48 ± 2.48	0.022*
8w	8.83 ± 2.67	8.57 ± 2.72	0.340
12 w	7.62 ± 2.51	7.72 ± 2.65	0.660
Follow-up period	7.52 ± 2.49	7.61 ± 2.38	0.745
Psychological state			
4w	4.26 ± 1.36	4.43 ± 1.45	0.650
8w	3.97 ± 1.36	4.14 ± 1.56	0.565
12w	3.51 ± 1.29	3.81 ± 1.39	0.086
Follow-up period	3.42 ± 1.24	3.72 ± 1.29	0.101
Functional status			
4w	5.08 ± 1.41	5.22 ± 1.52	0.697
8w	4.61 ± 1.58	4.55 ± 1.63	0.442
12 w	3.23 ± 1.36	4.04 ± 1.59	*P* < 0.001*
Follow-up period	2.71 ± 1.16	3.77 ± 1.46	*P* < 0.001*

*P < 0.05 for data comparison between groups aftertreatment

### SF-36

After 12 weeks’ treatment, most of the sub-dimensions of the SF-36 scores have improved in both groups. However, no significant differences were observed between the two groups (*P* > 0.05) (Table 5).

**Table 5:** Comparison of the dimension scores of SF-36 (FAS)

Dimension	Treatment group	Control group	*P* value
PF	92.99 ± 9.96	93.972 ± 9.18	0.268
RP	74.00 ± 34.36	74.44 ± 34.75	0.648
BP	71.85 ± 16.90	73.87 ± 17.17	0.171
GH	67.55 ± 15.71	66.70 ± 16.54	0.381
VT	75.96 ± 13.80	76.03 ± 14.48	0.892
SF	81.85 ± 14.59	83.40 ± 15.17	0.127
RE	78.72 ± 35.07	81.29 ± 32.52	0.174
MH	75.66 ± 13.54	74.24 ± 14.39	0.407
MCS	78.05 ± 16.18	78.74 ± 16.02	0.299
PCS	78.57 ± 13.61	78.03 ± 15.12	0.594

### Weight changes

Notably, we found patients taking flunarizine had a weight gain, whereas those taking Zhengtian Capsule were not evident. A significant difference between the two groups was observed in the change of weight gain from baseline and week 8 (*P* < 0.001), week 12 (*P* < 0.001) (Table 6).

**Table 6:** Comparison of weight changes (FAS)

Weight	Treatment group (*n* = 177)	Control group (*n* = 180)	*P* value
4w	59.88 ± 9.17	61.15 ± 10.01	0.290
8w	59.89 ± 9.13	61.51 ± 9.99	*P* < 0.001
12 w	60.27 ± 9.08	61.97 ± 10.00	*P* < 0.001
Follow-up period	60.31 ± 9.05	61.46 ± 10.07	0.871

### Safety and tolerability

In total, there were 46 (12.8 %) patients experienced adverse events in the process of the trial, 21 (11.86 %) from the treatment group, 25 (13.89 %) from the control group (*P* = 0.636). The adverse events were drowsiness, canker sore, upper respiratory tract infection, elevated ALT, et al. There was no need of special clinical dispose. No significant serious adverse events appeared in both groups. In addition, the compliance was similar in two groups and there was no statistical difference (*P* = 0.654).

## Discussion

In the present randomized controlled trial, Zhengtian Capsule and flunarizine were compared with respect to effectiveness and safety profile. We have shown that Zhengtian Capsule was non-inferior to flunarizine in responder rate at week 12 and follow-up period.

To our knowledge, this was the first active-controlled randomized trial of Zhengtian Capsule on migraine prevention which was consistent with Guidelines for Controlled Trials of Drugs in Migraine (second edition) [21]. In this trial, Zhengtian Capsule showed similar efficacy and safety with flunarizine, which was being recommended as a first-line drug for preventive treatment of migraine by European Federation of Neurological Societies (EFNS) [30].

Previous studies have shown that migraineurs accordantly report a lower quality of life in physical health, mental health, social functioning and academic performance compared with those non-migraineurs [31]. A large percent of migraine patients were presented functional symptoms [32] and associated somatic symptoms [33]. Negative affect is a common trigger of migraine which plays an important role in the occurrence and development of migraine [34]. In our trial, Zhengtian Capsule was found to have a strength in improving patients’ functional status and somatization symptoms thus improving the life quality of migraineurs.

Although there was no significant difference between the two groups regarding the incidence of adverse events in the current study and no severe adverse events occurred, there was unfavorable gained weight in the flunarizine group in our findings. This result was in agreement with the findings of previous trials by Sørensen PS [35], Luo N [36] and Wang LP [37]. Zhengtian Capsule, on the other hand, was not significantly affect body weight, complete blood count, or liver and kidney functions which suggested that this herbal drug is safe to use. Although the results of the present study need to be confirmed in future larger clinical trials, Zhengtian Capsule holds promising potential as an effective and practical means to prevent migraine.

The strengths of our study lies in the following aspects. Firstly, the sample size was large and patients’ selection biases were avoided with the randomization and blinding method. Secondly, in view of ethic we included an active control group instead of placebo control. Additionally, the design was consistent with Guidelines for Controlled Trials of Drugs in Migraine (second edition) and the outcome measures we used were reliable. Nevertheless, the present study has some limitations that warrant consideration. Most of the outcome measures used in our trial were based on patient’s subjective feelings and there weren’t many objective indicators. Besides, a daily dose of 5 mg flunarizine was a relatively low dose compared with the previous studies [38, 39] which may have led to a modest improvement in the efficacy outcome measures of migraine. Ten milligram flunarizine may guarantee a more rapid efficacy [40]. Therefore, we should bear these in mind when interpreting our findings.

## Conclusion

In summary, Zhengtian Capsule was non-inferior to flunarizine with regard to the primary endpoint. In addition, it could reduce migraine days and improve the functional status and somatization symptoms of migraine patients with good safety profile. This Chinese Patent Medicine may therefore be an option for the prophylactic treatment of migraine.

## Abbreviations

ACEI: Angiotensin converting enzyme inhibitor
AEDs: Antiepileptic drugs
ANCOVA: Analysis of covariance
ARB: Angiotensin receptor blockers
BP: Body pain
CCB: Calcium channel blockers
CPM: Chinese patent medicine
CRF: Case Report Form
EFNS: European Federation of Neurological Societies
GCP: Good clinical practice
GH: General Health
LOCF: Last observation carried forward
MA: Migraine with a typical aura
MCS: Mental component summary
MH: Mental health
MO: Migraine without aura
PCS: Physical component summary
PF: Physical functioning
PRO: Patient-reported outcome
Qn: Quaque nocte
RE: Role emotional
RP: Role physical
SF: Social functioning
SF-36: Short-form health survey scale
Tid: Three times a day
VAS: Visual analog scale
VT: Vitality
